# Identification and validation of a novel six-gene signature based on mucinous adenocarcinoma-related gene molecular typing in colorectal cancer

**DOI:** 10.1007/s12672-024-00916-2

**Published:** 2024-03-05

**Authors:** Yuxin Man, Dao Xin, Yang Ji, Yang Liu, Lingna Kou, Lingxi Jiang

**Affiliations:** 1 Department of Medical Oncology, Sichuan Clinical Research Center for Cancer, Sichuan Cancer Hospital & Institute, Sichuan Cancer Center, Affiliated Cancer Hospital of University of Electronic Science and Technology of China, Chengdu, China; 2 Department of Oncology, The First Affiliated Hospital of Zhengzhou University, Zhengzhou, China; 3 School of Medicine, University of Electronic Science and Technology of China, Chengdu, China; 4 Sichuan Provincial Key Laboratory for Human Disease Gene Study, Department of Laboratory Medicine, Sichuan Provincial People’s Hospital, University of Electronic Science and Technology of China, Chengdu, China

**Keywords:** Colorectal cancer, Mucinous adenocarcinoma, Gene, Signature, Survival

## Abstract

**Background and objectives:**

Colorectal mucinous adenocarcinoma (MAC) is a particular pathological type that has yet to be thoroughly studied. This study aims to investigate the characteristics of colorectal MAC-related genes in colorectal cancer (CRC), explore the role of MAC-related genes in accurately classifying CRC, and further construct a prognostic signature.

**Methods:**

CRC samples were collected from The Cancer Genome Atlas (TCGA) and Gene Expression Omnibus (GEO). MAC-related differentially expressed genes (DEGs) were analyzed in TCGA samples. Based on colorectal MAC-related genes, TCGA CRC samples were molecularly typed by the non-negative matrix factorization (NMF). According to the molecular subtype characteristics, the RiskScore signature was constructed through univariate Cox, the least absolute shrinkage and selection operator (LASSO), and multivariate Cox regression analyses. Clinical significance in CRC of the RiskScore signature was analyzed. A nomogram was further built based on the RiskScore signature.

**Results:**

From the colorectal MAC-related genes, three distinct molecular subtypes were identified. A RiskScore signature composed of six CRC subtype-related genes (*CALB1*, *MMP1*, *HOXC6*, *ZIC2*, *SFTA2,* and *HYAL1*) was constructed. Patients with high-RiskScores had the worse prognoses. RiskScores led to differences in gene mutation characteristics, antitumor drug sensitivity, and tumor microenvironment of CRC. A nomogram based on the signature was developed to predict the one-, three-, and five-year survival of CRC patients.

**Conclusion:**

MAC-related genes were able to classify CRC. A RiskScore signature based on the colorectal MAC-related molecular subtype was constructed, which had important clinical significance for guiding the accurate stratification of CRC patients.

## Introduction

The global incidence and mortality of colorectal cancer (CRC) are among the top three malignant tumors [1]. Approximately 20% of CRC patients have metastases when they are diagnosed, and 50% of patients with localized disease will ultimately develop metastases [2]. However, the five-year survival rate of metastatic CRC is just 14% [3]. It is one of the most severe gastrointestinal tumor diseases that seriously threaten the lives and health of people.

The occurrence and development of CRC is a complex and heterogeneous process involving a variety of molecules and signal pathways [2]. The classification and refinement of CRC according to histological and molecular biological characteristics has been the embodiment of precision treatment [4, 5]. The histological type of CRC profoundly affects its clinical characteristics and outcomes [6–8]. However, the clinical practice remains challenging, and many patients are insensitive to treatment and have relatively poor prognoses.

Notably, mucinous adenocarcinoma (MAC) is a specific histopathological subtype of CRC, and it is characterized by abundant extracellular mucin in more than 50% of the tumor tissue [9, 10], with an incidence of 10–15% [11, 12]. Cancer cells in colorectal MAC have goblet cell characteristics [13] and produce large amounts of mucin glycoproteins [14]. In colorectal MAC, MUC1 and MUC13 are transmembrane mucins, while MUC2 and MUC5AC are secreted gell-forming mucins resistant to tumor cell death and form an immune infiltrating barrier [12]. In comparison to non-mucinous adenocarcinoma (NMAC), MAC is more common in women, and it has more advanced T or N stage, proximal location, worse differentiation, and a higher ratio of peritoneal implant, microsatellite instability-H (MSI-H) or *BRAF* mutation [15–18]. Due to the inherent characteristics of MAC, it often responds poorly to conventional therapy [14, 19, 20]. MAC often lacks the attention of clinical practice guidelines and faces a more complex treatment situation.

In comparison with NMAC, the unique clinical and pathological features of MAC indicate that there are some characteristic genes in MAC that can be stratified for evaluating patients and guiding clinical treatment. However, the significance of the typical genes and the prognostic factors of colorectal MAC yet remain to be determined, and relatively few studies have been conducted in this area. A comprehensive analysis is essential for elucidating the potential mechanism of MAC for proper treatment.

**Fig. 1:**
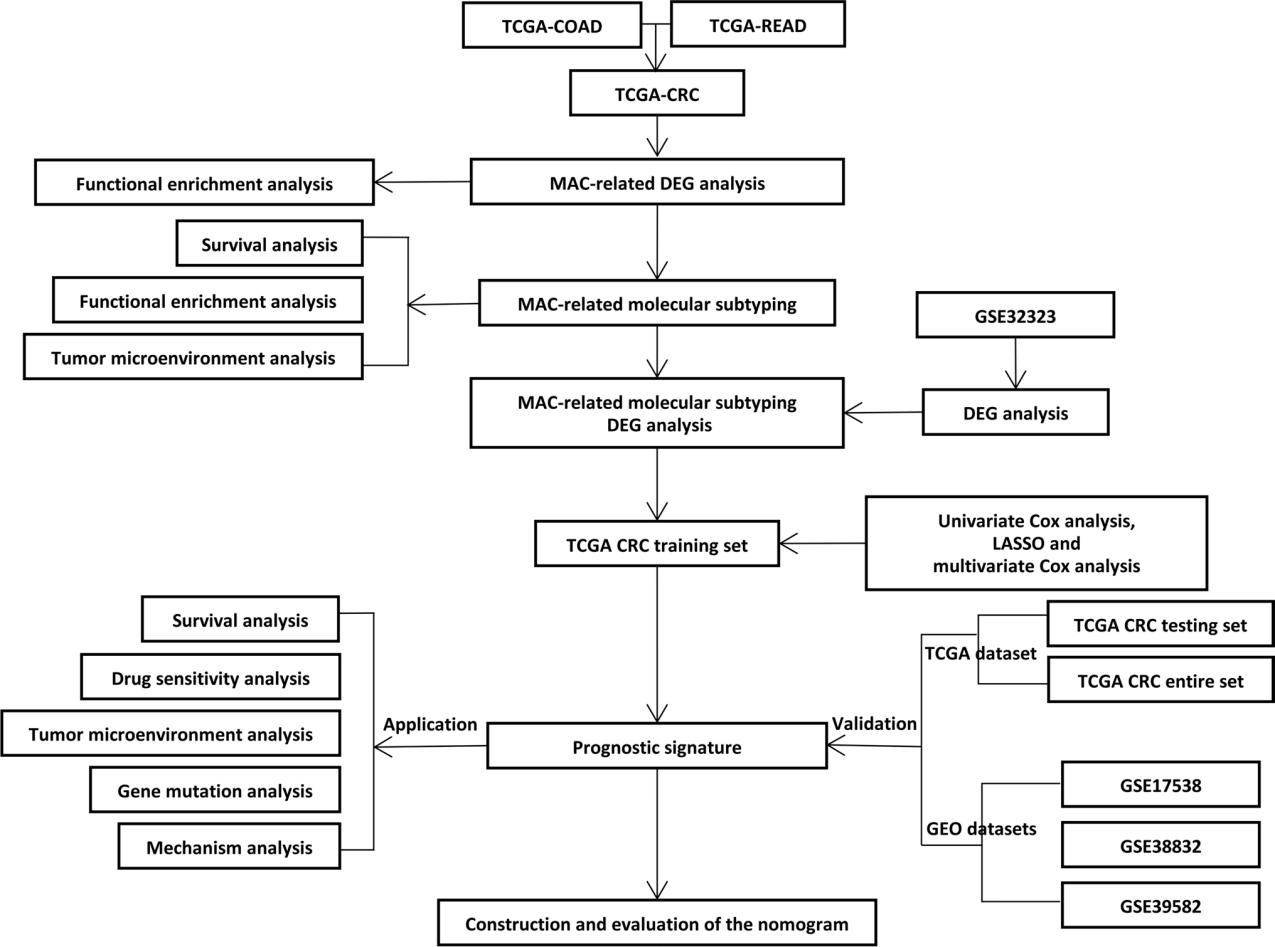
Study flow chart

In the era of big data, valuable features in data mining and analysis can improve the accuracy of decisions made by clinicians. Sequencing data can be obtained from multiple databases, and various bioinformatics analyses can be performed, which helps gain a more comprehensive and accurate understanding of MAC. In this study, differentially expressed genes (DEGs) were initially analyzed in colorectal MAC. Based on colorectal MAC-related DEGs, the molecular subtypes of CRC were classified using the non-negative matrix factorization (NMF) method. The clinical and prognostic characteristics of different subtypes were then analyzed. The gene expression differences were analyzed between the subtypes with the most significant differences in clinical characteristics. In combination with the genes closely related to the occurrence and development of CRC, univariate Cox, the least absolute shrinkage and selection operator (LASSO), and multivariate Cox regression were used for constructing the CRC molecular typing-related prognosis signature in CRC. Multiple datasets were used for verifying the signature, and the clinical characteristics and molecular differences between the high- and low-RiskScore groups were identified. A nomogram was ultimately drawn for predicting the survival of CRC patients. CRC molecular typing-related risk signature can serve as a potential genetic marker and prognostic classification basis for CRC. The workflow of this study can be seen in Fig. 1.

## Materials and methods

### Data acquisition and DEG analysis

TCGA-COAD and TCGA-READ datasets were downloaded using the TCGAbiolinks [21] package. CRC-related datasets, including GSE39582 [22], GSE17538 [23], GSE38832 [24], and GSE32323 [25], were downloaded from the GEO database using the GEOquery [26] package. The DESeq2 package [27] was used for analyzing DEGs in the RNA-seq data. The Limma [28] package was used to analyze the DEGs in the microarray data. DEGs in the RNA-seq data were defined as the adjusted *P* value < 0.05 and | log_2_foldchange |> 1. DEGs in the microarray data were defined as the *P* value < 0.05 and | log_2_foldchange |> 1. Maftools package [29] was used to visualize TCGA-COAD and TCGA-READ gene mutation data.

### Molecular typing based on MAC-related genes

MAC-related genes were included for molecular typing analysis of TCGA CRC samples by the NMF [30] method. The optimal number of clusters was determined by cophenetic correlation, dispersion, and silhouette.

### Construction of a prognostic signature and survival analysis

The *P* < 0.05 was used as the threshold, and the univariate Cox regression analysis was used for detecting the critical genes related to molecular typing of CRC in the training set. In order to narrow the gene range and maximize the accuracy, the LASSO regression was used to identify prognostic genes using the glmnet package [31]. Multivariate Cox regression analysis was used to establish the prognostic RiskScore signature, and the RiskScore was calculated.

$$RiskScore = {{h}_{0}\left(t\right){\text{exp}}\sum }_{j=1}^{n}Coefj\times Xj$$

The area under the curve (AUC) of the receiver operating characteristic (ROC) curve was used for quantifying the prediction ability of the signature through the R package timeROC [32]. Kaplan–Meier survival curve was used to describe differences in survival time among patients with different RiskScores.

### Functional enrichment analysis

The clusterProfiler [33] package was used for gene ontology (GO) analysis [34] and Kyoto encyclopedia of genes and genomes (KEGG) [35] enrichment analysis. The “h.all.v7.4.symbols.gmt” gene sets were downloaded from the MSigDB database [36] for gene set enrichment analysis (GSEA), and the gene set variation analysis (GSVA) package was used for identifying the hallmark gene set score.

### Estimation of tumor-infiltrating immune cells and analysis of IC_50_ values of common antitumor drugs

ImmuneScore, StromaScore, and ESTIMATEScore were analyzed using the estimate [37] package based on gene expression data. CIBERSORTx (https://cibersortx.stanford.edu/) [38] and ImmuneCellAI online tools (http://bioinfo.life.hust.edu.cn/ImmuCellAI#! /) [39] were used for predicting the degree of tumor-infiltrating immune cells in the tumor microenvironment of samples. The pRRophetic package [40] was used to predict the half-maximal inhibitory concentration (IC_50_) values of antitumor drugs based on the sample gene expression data.

### Construction of the nomogram

A nomogram was constructed to predict CRC patients' overall survival (OS) rates at one, three, and five years. We also built calibration curves to evaluate the difference between the OS rate predicted by the nomogram and the observed OS rate. We used decision curve analysis (DCA) to assess the clinical practicability of the nomogram.

### Statistical analysis

R v. 4.1.2 was used for statistical analysis. Student’s *t-*test or Wilcoxon test was used to establish the difference between the two groups. Multiple groups were compared using one-way ANOVA or the Kruskal–Wallis test. The chi-square test was used for comparison of frequencies. *P* < 0.05 was considered to be statistically significant.

## Results

### Analysis of MAC-related genes in CRC

**Fig. 2:**
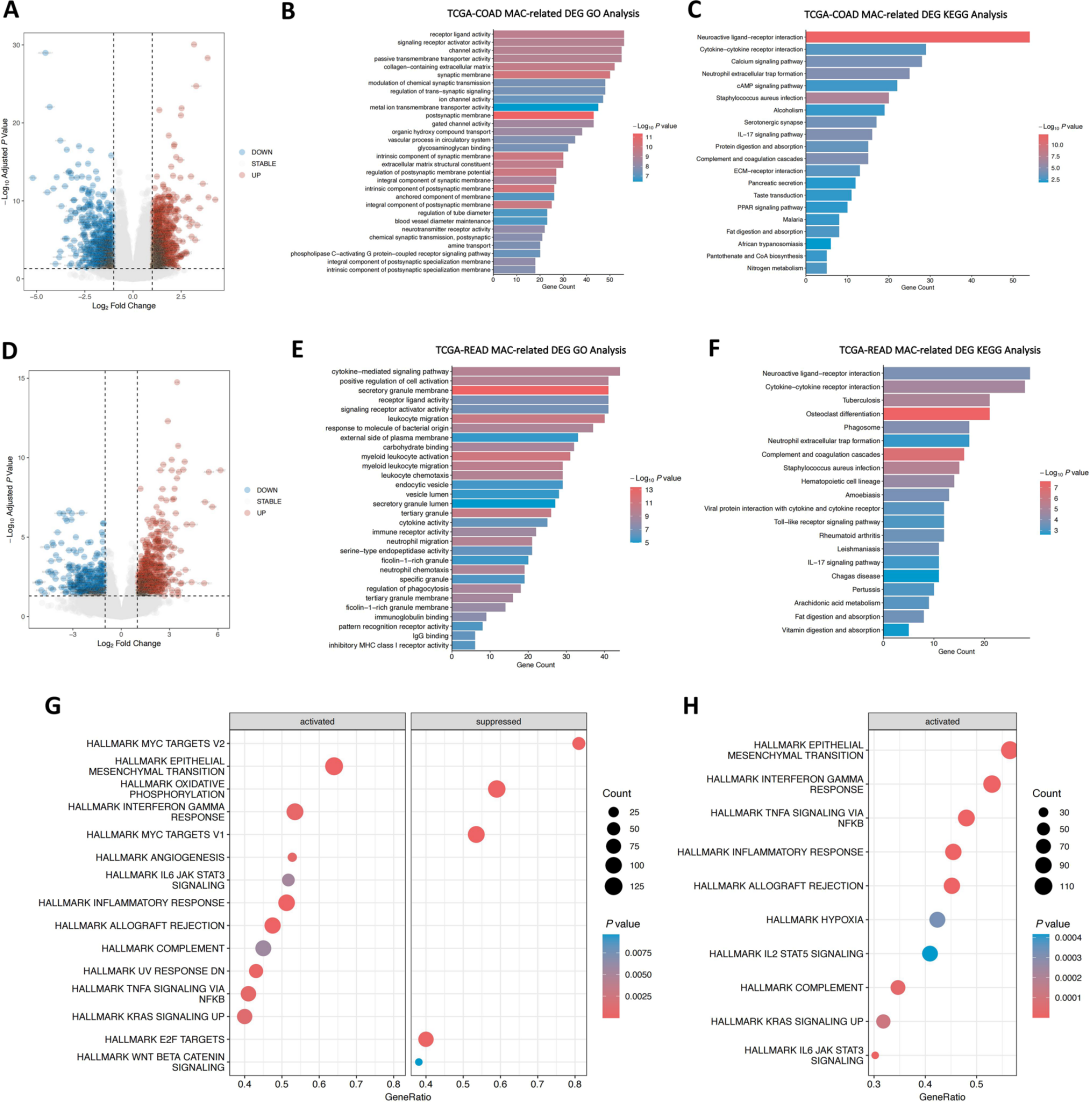
Analysis of the MAC-related genes. **A** Volcano plot of MAC-related DEGs in the TCGA-COAD dataset. **B** GO analysis of MAC-related DEGs in the TCGA-COAD dataset. **C** KEGG analysis of MAC-related DEGs in the TCGA-COAD dataset. **D** Volcano plot of MAC-related DEGs in the TCGA-READ dataset. **E** GO analysis of MAC-related DEGs in the TCGA-READ dataset. **F** KEGG analysis of MAC-related DEGs in the TCGA-READ dataset. **G** GSEA of MAC-related genes in the TCGA-COAD dataset. **H** GSEA of MAC-related genes in the TCGA-READ dataset

In comparison to the small number of normal samples, more MAC samples for subsequent analysis were found in the TCGA database. We analyzed DEGs between 58 MAC samples and 328 adenocarcinoma samples in the TCGA-COAD dataset. DEGs were analyzed between 14 MAC samples and 118 adenocarcinoma samples in the TCGA-READ dataset. DEGs were presented in volcano plots (Fig. 2A and D). There were 1418 differentially up-regulated genes and 736 differentially down-regulated genes in TCGA-COAD MAC. A total of 606 genes were up-regulated and 372 down-regulated in TCGA-READ MAC. We further performed GO and KEGG analyses on MAC-related DEGs to explore the potential functional mechanism (Fig. 2B, C, E, and F). In CRC, according to the results of GO analysis, MAC-related DEGs were involved in receptor-ligand activity, signaling receptor activator activity, and other functional mechanisms. According to the results of KEGG analysis, MAC-related DEGs were involved in neuroactive ligand-receptor interaction, cytokine-cytokine receptor interaction, and IL-17 signaling pathway. Our results suggested that DEGs were closely related to the malignant biological behavior of colorectal MAC. GSEA of the hallmark gene set was performed on the MAC-related genes, which indicated that the epithelial-mesenchymal transition (EMT) pathway-related gene set was activated (Fig. 2G and H).

### Identification of molecular subtypes in CRC

**Fig. 3:**
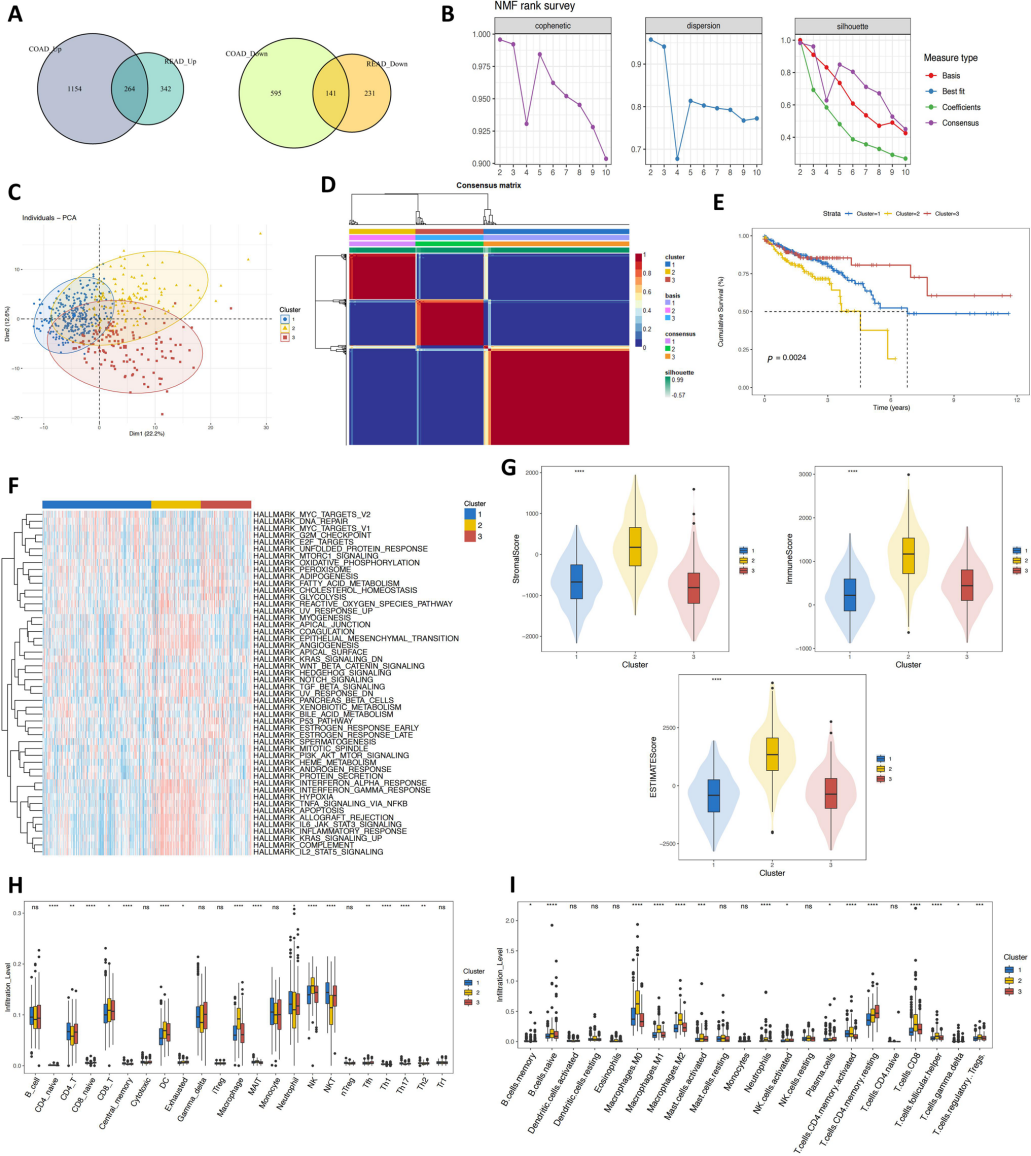
Molecular subtyping analysis of MAC-related genes in CRC. **A** Venn diagram of the intersection of TCGA colorectal MAC-related genes. **B** Line charts of phenotypic correlation, dispersion, and silhouette at rank = 2–10. **C** PCA graph of different subtypes of TCGA CRC dataset. **D** Consensus graph of NMF clustering. **E** Survival analysis of three molecular subtypes. **F** GSVA heatmap of three molecular subtypes. **G** ESTIMATE analysis of three molecular subtypes. **H** ImmuCellAI analysis of three molecular subtypes. **I** CIBERSORTx analysis of three molecular subtypes. ****P < 0.0001; ***P < 0.001; **P < 0.01; *P < 0.05; ns: no significance

The intersection of genes related to TCGA-COAD MAC and TCGA-READ MAC showed 264 genes to be up-regulated and 141 genes to be down-regulated simultaneously. A total of 405 genes that may have biological importance for colorectal MAC were identified (Fig. 3A). Survival analysis was performed on the critical genes grouped based on the optimal truncation value of patient survival. Genes with *P* < 0.05 were selected, and 192 MAC-related key genes were found to have a correlation with the OS of CRC patients in the TCGA dataset. These 192 MAC-related key genes were then used for the molecular typing of CRC by the NMF method. According to phenotype, dispersion, and silhouette, the optimal number of clusters was three (Fig. 3B). Three molecular subtypes were obtained: Cluster 1 (n = 280), Cluster 2 (n = 127), and Cluster 3 (n = 130). The PCA diagram (Fig. 3C) showed an apparent distinction among CRC's molecular subtypes, indicating that the three molecular subtypes were well differentiated. The consistency clustering heatmap can be seen in Fig. 3D. Survival analysis was performed for samples with survival data, and the Kaplan–Meier curves showed significant differences in the survival of patients with different molecular subtypes (Fig. 3E).

GSVA were then performed on the tumor-related gene sets “H.all.v7.4.symbols.gmt” of three molecular subtypes, and a heatmap was drawn for the GSVA scores (Fig. 3F). From the heatmap, it can be found that “EPITHELIAL_MESENCHYMAL_TRANSITION”, “INFLAMMATORY_RESPONSE” or “ANGIOGENESIS” got relatively higher scores in Cluster 2. In addition, the differences in immune characteristics among the three subtypes were analyzed. The molecular subtypes' ImmuneScore, StromalScore, and ESTIMATEScore were all calculated (Fig. 3G). Scores in Cluster 2 were relatively high, which indicated more immune and stromal components in the tumor microenvironment. Therefore, the degree of cell invasion in the tumor microenvironment of the three molecular subtypes was further explored. Significant differences in infiltrating cells were observed in ImmuCellAI (Fig. 3H) and CIBERSORTx (Fig. 3I) analyses. The degree of macrophage invasion in the tumor microenvironment of Cluster 2 was higher, and NK-T cell infiltration was considerably lower. These results suggested that the three molecular subtypes of CRC differentiated by MAC-related genes have significant differences in clinical prognosis and potential mechanism pathways, with apparent differentiation.

### Identification of CRC molecular typing-related genes

Significant biological differences were observed in the three molecular subtypes distinguished by the key genes of MAC, from clinical features to mechanism. Therefore, DEGs between Cluster 2 and Cluster 1 and DEGs between Cluster 2 and Cluster 3 were analyzed. 3,733 genes were up-regulated, and 1,009 genes were down-regulated between Cluster 2 and Cluster 1. 2,674 genes were up-regulated, and 833 genes were down-regulated between Cluster 2 and Cluster 3.

**Fig. 4:**
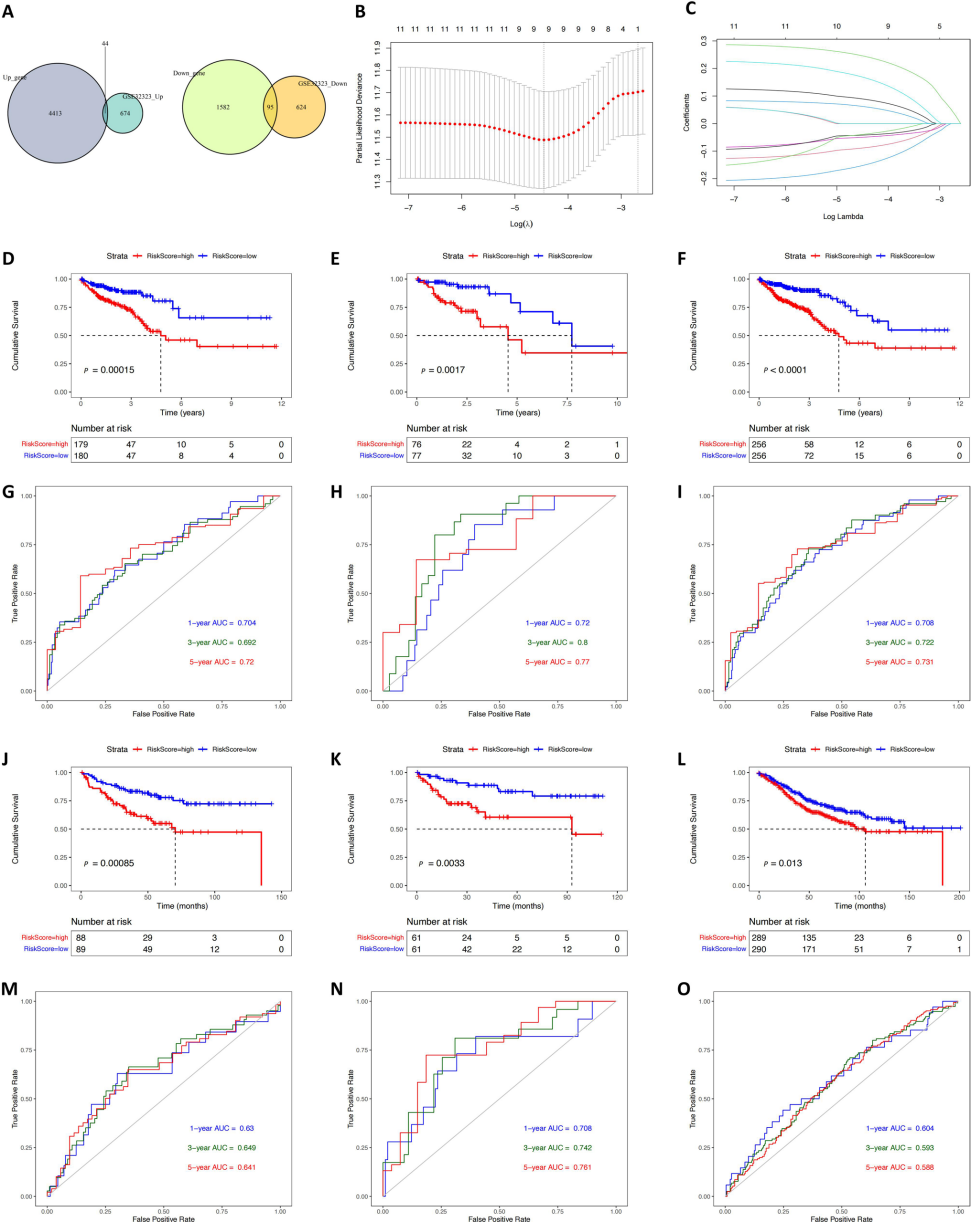
Construction and validation of the signature. **A** Venn diagram of the intersection of key genes in CRC samples. **B** Ten-fold cross-validation of lambda selection by LASSO. **C** The LASSO coefficient spectrum in the TCGA training set. **D**–**F** Survival analysis showed the difference in the prognoses of patients in the TCGA CRC training set, internal testing set, and entire set. **G**–**I** The one-, three-, and five-year ROC curves of the TCGA CRC training set, internal testing set, and entire set. **J**–**L** Kaplan–Meier curves in GSE17538, GSE38832, and GSE39582. **M**–**O** The one-, three-, and five-year ROC curves in GSE17538, GSE38832, and GSE39582

After merging the two groups of DEGs, 4,457 were up-regulated, and 1,677 were down-regulated. Due to the large number of genes obtained by MAC-related molecular typing, we analyzed the DEGs of 17 paired CRC samples in the GSE32323 dataset from the GEO database to narrow the gene range. Then, we screened out genes which were closely associated with the occurrence and development of CRC. The prognostic signature could be constructed in the whole range of CRC, and the applicability of the prognostic signature could be expanded. In the GSE32323 dataset, 718 genes were up-regulated, and 719 genes were down-regulated. There were 139 genes in the intersection of up- and down-regulated genes (Fig. 4A). This gene set may play an essential role in CRC.

### Development of the RiskScore signature

The gene set that contained 139 genes was obtained using the aforementioned steps. After removing samples with missing survival data or the survival time of 0, the entire TCGA CRC set (512 samples) was randomly divided into the training set and the internal testing set (7:3). Based on the expression levels of 139 CRC molecular typing-related genes and survival information in the training set, we constructed a CRC prognostic signature. Firstly, *P* < 0.05 was chosen as the screening threshold, and univariate Cox analysis was performed on the 139 genes in the training set to screen out the genes with prognostic significance. A total of 12 genes met the screening threshold by univariate Cox analysis. Since the number of 12 genes was still relatively large, we further compressed these 12 genes using the LASSO regression (Fig. 4B and C). Ten-fold cross-validation was performed in the LASSO regression. When lambda = 0.0116, 12 genes were compressed to nine genes. Therefore, these nine genes were included in the multivariate Cox analysis, and the stepwise method was used to construct the signature.

The six genes found to be related to CRC molecular typing were ultimately used for constructing a prognostic signature of CRC patients: *CALB1*, *MMP1*, *HOXC6*, *ZIC2*, *SFTA2*, and *HYAL1*. The following formula was used for calculating the RiskScore (the coefficients were rounded to seven decimal places).

$$RiskScore = {h}_{0}\left(t\right){\text{exp}}(0.1187114\times CALB1-0.1208561\times MMP1+0.3187824\times HOXC6+0.0974445\times ZIC2+0.2239488\times SFTA2-0.2384973\times HYAL1)$$

The samples were divided into high- and low-RiskScore groups based on the median RiskScore in the TCGA training set, TCGA internal testing set, and TCGA entire set. Kaplan–Meier analysis suggested that low-RiskScore patients had a significantly better survival outcome. ROC curve results showed the signature constructed in this study had the good predictive ability (five-year AUC = 0.720; three-year AUC = 0.692; one-year AUC = 0.704). In particular, the AUC values of the TCGA internal testing set and the entire set at one, three, and five years were all greater than 0.7, and the AUC value of the internal testing set at three years was 0.8. Therefore, it could be seen that this prognostic signature showed good discriminative and predictive ability (Fig. 4D-I). To determine the independence of the six-gene signature in clinical application, univariate and multivariate Cox regression analyses were conducted on clinical variables in TCGA CRC samples with complete clinical information (shown in Table 1). Univariate Cox regression analysis showed that stage III/IV vs. stage I/II, age, and the RiskScore were significantly correlated with survival. However, gender was not a prognostic factor. In multivariate Cox analysis, stage III/IV vs. stage I/II, age, and the RiskScore had a significant correlation with survival. These results suggested the potential for the six-gene signature to be used as an independent risk factor for predicting the prognosis of CRC patients.

**Table 1:** Univariate and multivariate Cox analyses

Variables	Univariable Cox analysis				Multivariable Cox analysis			
	HR	95% CI of HR		*P*	HR	95% CI of HR		*P*
		lower	upper			lower	upper	
Age	1.04	1.02	1.06	< 0.01	1.04	1.02	1.06	< 0.01
Gender	0.92	0.60	1.41	0.70	–	–	–	–
Stage	3.30	2.09	5.21	< 0.01	3.35	2.10	5.33	< 0.01
RiskScore	1.45	1.31	1.61	< 0.01	1.33	1.19	1.48	< 0.01

### Validation of the six-gene signature in three independent GEO CRC datasets

Three independent GEO datasets (GSE17538, GSE38832, and GSE39582) were used as external testing sets for verifying the prognostic signature. The samples were divided into high- and low-RiskScore groups based on the median RiskScore of each dataset. In the external testing sets, Kaplan–Meier curves showed the median OS of patients in the high-RiskScore group to be significantly shorter than in the low-RiskScore group (Fig. 4J, K, and L). The one-, three-, and five-year AUC values also suggested that the prognostic signature had the good predictive ability in the three independent datasets (Fig. 4M, N, and O).

### Correlation between RiskScores and clinicopathological features

**Fig. 5:**
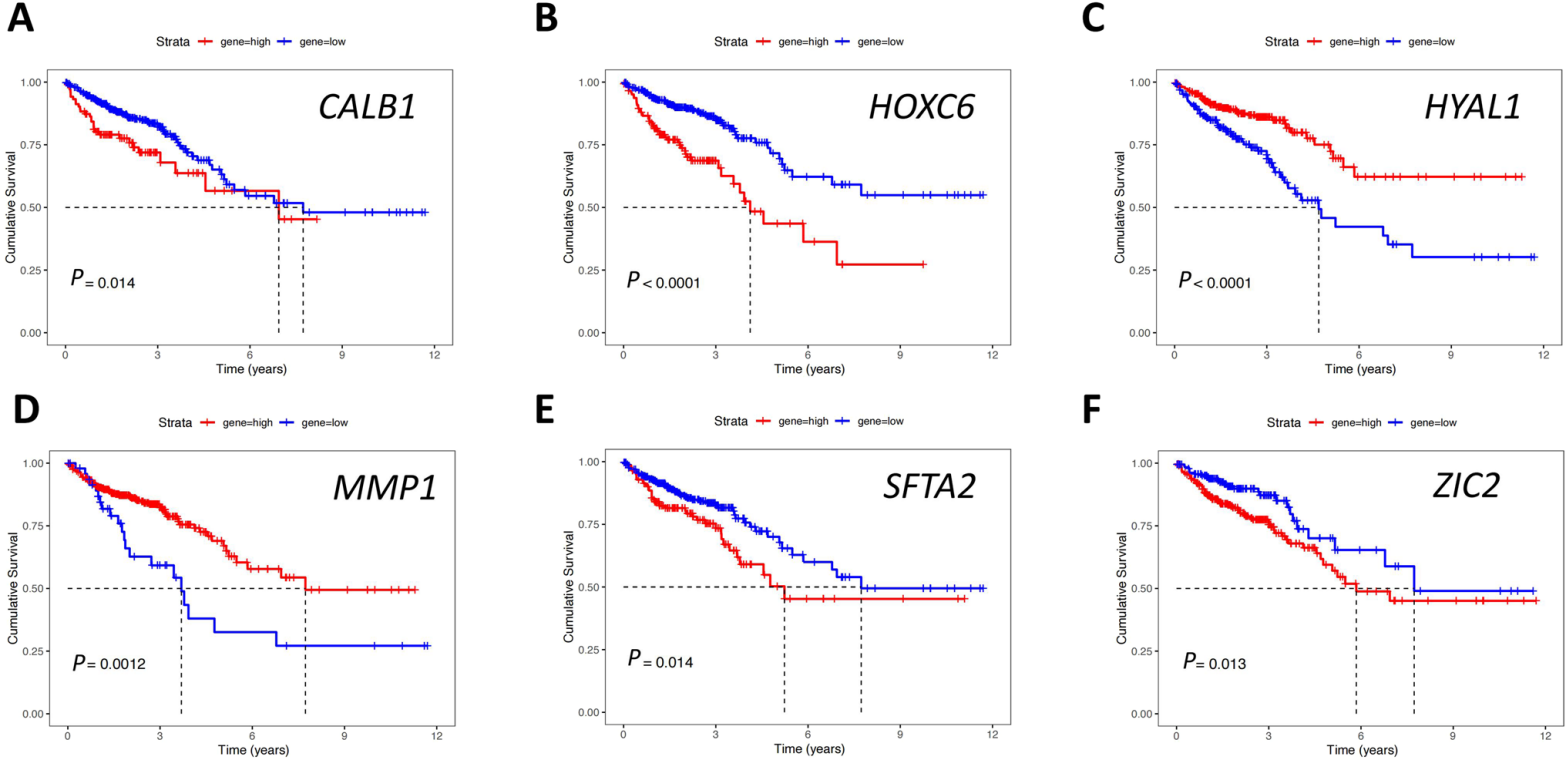
The effect of signature genes on the prognosis of patients in the TCGA CRC dataset. **A** Effect of *CALB1* on patient survival. **B** Effect of *HOXC6* on patient survival. **C** Effect of *HYAL1* on patient survival. **D** Effect of *MMP1* on patient survival. **E** Effect of *SFTA2* on patient survival*.* **F** Effect of *ZIC2* on patient survival

**Fig. 6:**
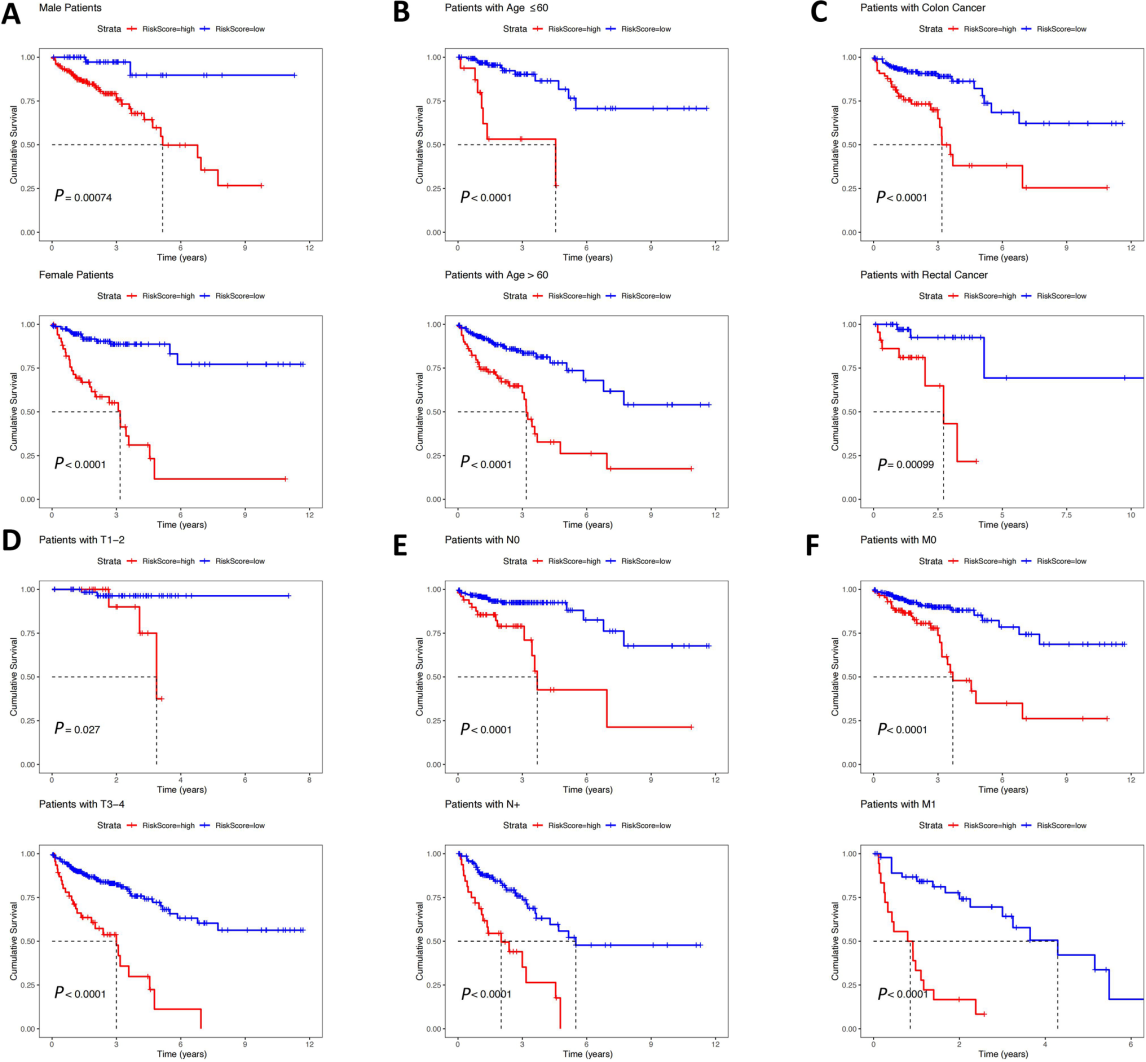
Survival analysis between RiskScores and different clinical parameters in the TCGA CRC dataset. **A** Effect of RiskScores on the prognosis of different gender. **B** Effect of RiskScores on the prognosis of different ages. **C** Effect of RiskScores on the prognosis of different primary tumor locations. **D** Effect of RiskScores on the prognosis of different T stages. **E** Effect of RiskScores on the prognosis of different N stages. **F** Effect of RiskScores on the prognosis of different M stages

Based on the best cut-off *P* values, we first analyzed the prognostic value of these six genes in the TCGA CRC dataset. The results indicated that patients with high expression of *CALB1*, *HOXC6*, *ZIC2,* or *SFTA2* had poor prognoses, while those with high expression of *MMP1* or *HYAL1* had good prognoses (Fig. 5A-F). It could be inferred that the six genes had a close association with the CRC prognosis. The relationship between the RiskScores and clinical parameters of CRC was further explored. Regardless of gender, age, tumor locations, or T/N/M stages, patients with low-RiskScores were found to have relatively better prognoses (Fig. 6A-F).

**Fig. 7:**
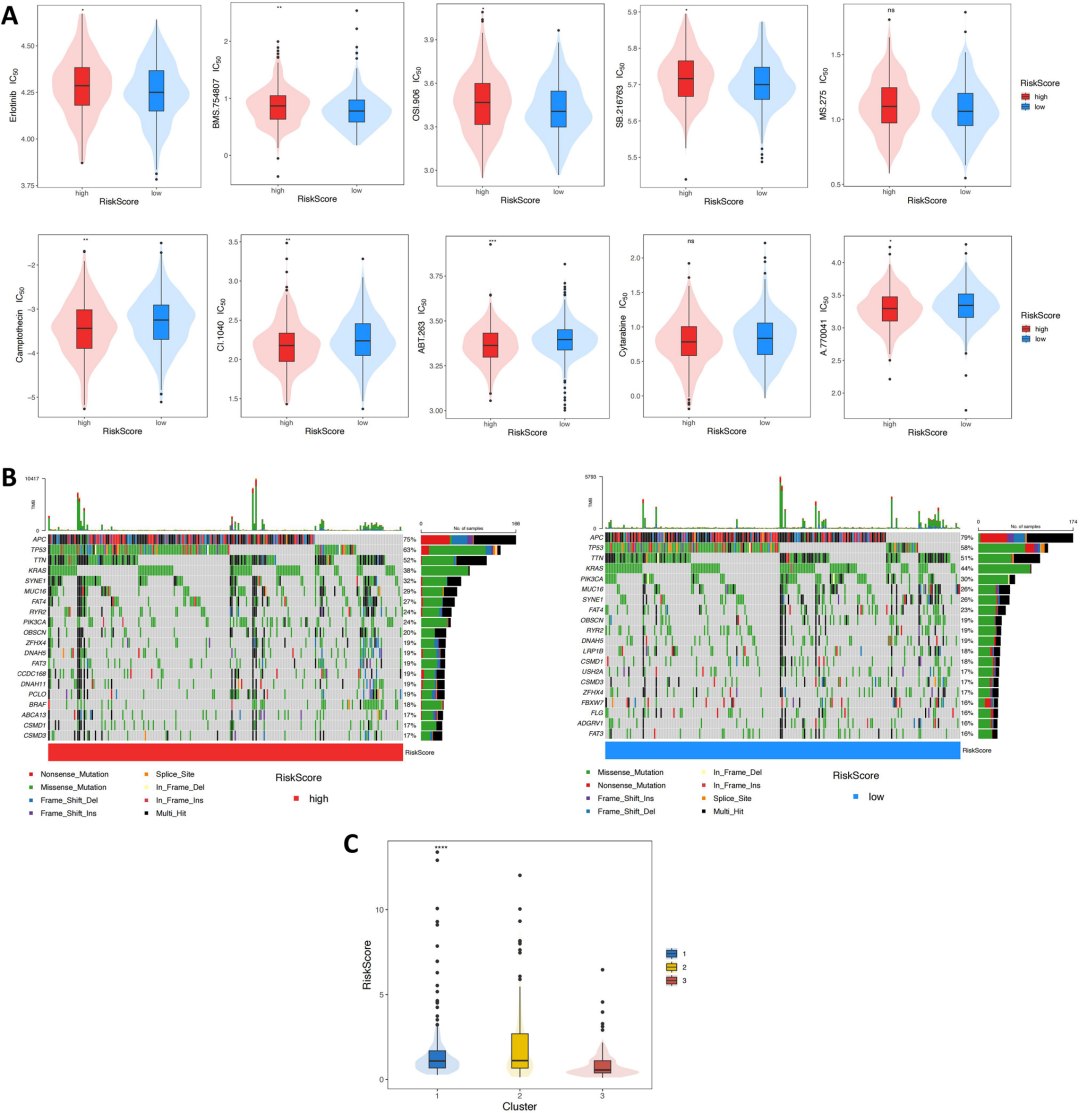
Analysis of clinical features of the signature. **A** IC_50_ values of antitumor drugs in patients with different RiskScores. **B** Gene mutation waterfall diagram of TCGA CRC samples with different RiskScores. **C** Relationship between different Clusters and RiskScores. ****P < 0.0001; ***P < 0.001; **P < 0.01; *P < 0.05; ns: no significance

Subsequently, we further analyzed the relationship between RiskScores and clinical characteristics in the aforementioned TCGA CRC entire set. The IC_50_ values of Erlotinib, BMS.754807, OSI.906, and SB.216763 in the low-RiskScore group were found to be lower. The corresponding IC_50_ values of Camptothecin, CI.1040, ABT.263, and A.770041 were all higher. No significance was found in the IC_50_ values of MS.275 and Cytarabine between the two groups (Fig. 7A). These results suggested that patients in the high-RiskScore group could choose drugs with lower corresponding IC_50_ values, potentially improving efficacy. The differences in gene mutations between high- and low-RiskScore groups were explored (Fig. 7B). The mutation rates in the high-RiskScore group of the first five were *APC* (75%), *TP53* (63%), *TTN* (52%), *KRAS* (38%), and *SYNE1* (32%). Mutation rates in the low-RiskScore group of the first five were *APC* (79%), *TP53* (58%), *TTN* (51%), *KRAS* (44%), and *PIK3CA* (30%). Interestingly, we found a significant difference in the distribution of *BRAF* mutation between the two groups (*P* < 0.001), with 18% in the high-RiskScore group and 6% in the low-RiskScore group. The relationship between RiskScores and three Clusters formed by MAC-related genes was further analyzed (Fig. 7C). We were also surprised that Cluster 3 with the low RiskScore had the best prognosis, while Cluster 2 with the high RiskScore had the worst prognosis.

### Evaluation of the tumor microenvironment in groups stratified by the RiskScore

**Fig. 8:**
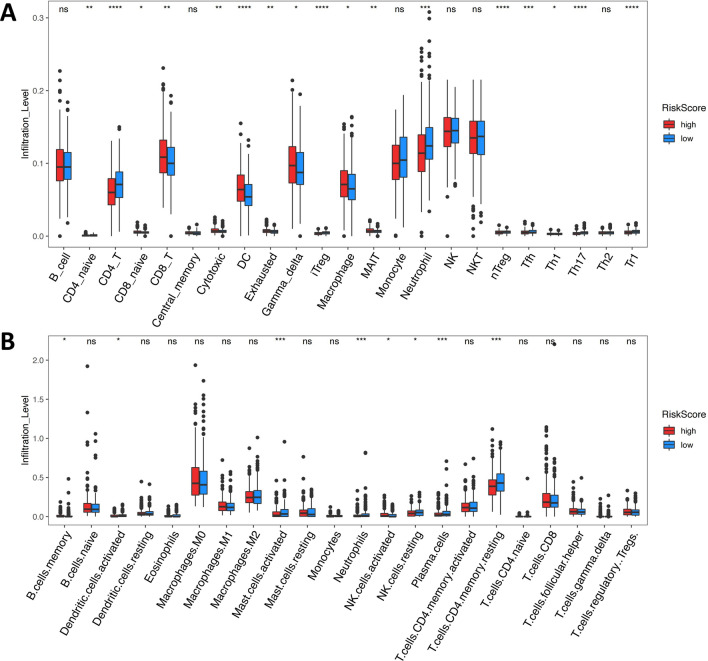
**A** ImmuCellAI analysis of tumor microenvironment immune infiltrating cells in different RiskScore groups. **B** CIBERSORTx analysis of tumor microenvironment immune infiltrating cells in different RiskScore groups. ^****^P < 0.0001; ^***^P < 0.001; ^**^P < 0.01; ^*^P < 0.05; ns: no significance

ImmuCellAI and CIBERSORTx online tools were used for analyzing the immune infiltrating cells of patients in the high- and low-RiskScore groups. A significant difference was observed in some immune infiltrating cells between the high- and low-RiskScore groups. ImmuCellAI analysis (Fig. 8A) showed that the infiltration levels of macrophages and DC were higher in the high-RiskScore group. CIBERSORTx analysis showed that the infiltration levels of plasma cells and activated mast cells were higher in the low-RiskScore group (Fig. 8B). Through the above analyses, it was found that the RiskScore had an impact on the tumor microenvironment of CRC samples.

### Analysis of the differences in molecular mechanism caused by the RiskScore

**Fig. 9:**
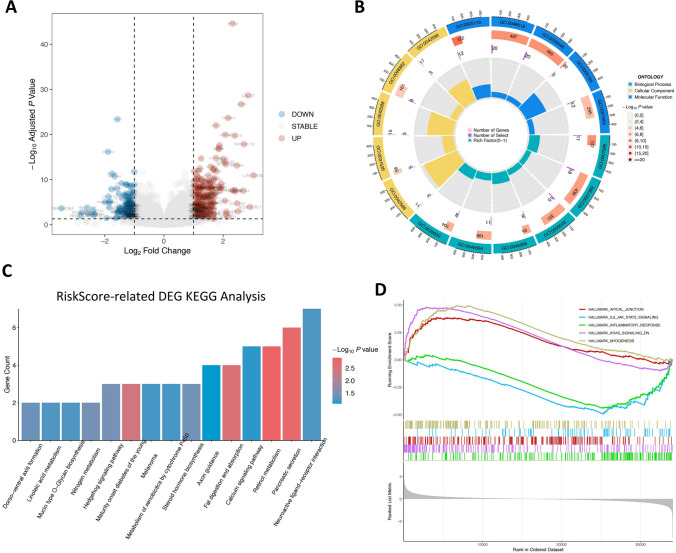
Functional enrichment analyses of the signature. **A** Volcano plot of DEGs caused by different RiskScores. **B** GO analysis of DEGs caused by different RiskScores. **C** KEGG analysis of DEGs caused by different RiskScores. **D** GSEA of RiskScore-related genes

Due to the significant differences in clinical characteristics caused by the RiskScore, DEGs in the high- and low-RiskScore groups in the aforementioned TCGA CRC entire dataset were further explored (Fig. 9A). GO analysis was conducted on DEGs (Fig. 9B). It was found that DEGs were mainly involved in the receptor ligand activity, signaling receptor activator activity, and some metabolic processes. KEGG analysis of the potential mechanisms conducted on DEGs (Fig. 9C) showed that DEGs caused by the RiskScore were engaged in tumor occurrence and development-related pathways and metabolic-related pathways. GSEA was then further performed on the two groups with different RiskScores, and the primary mechanism caused by the RiskScore was analyzed using hallmark gene sets (Fig. 9D). It could be seen that HALLMARK_APICAL_JUNCTION, HALLMARK_MYOGENESIS, and HALLMARK_KRAS_SIGNALING_DN were activated, while HALLMARK_INFLAMMATORY_RESPONSE and HALLMARK_IL6_JAK_STAT3_SIGNALING were inhibited.

### Construction of a nomogram combined with the RiskScore and clinical features

**Fig. 10:**
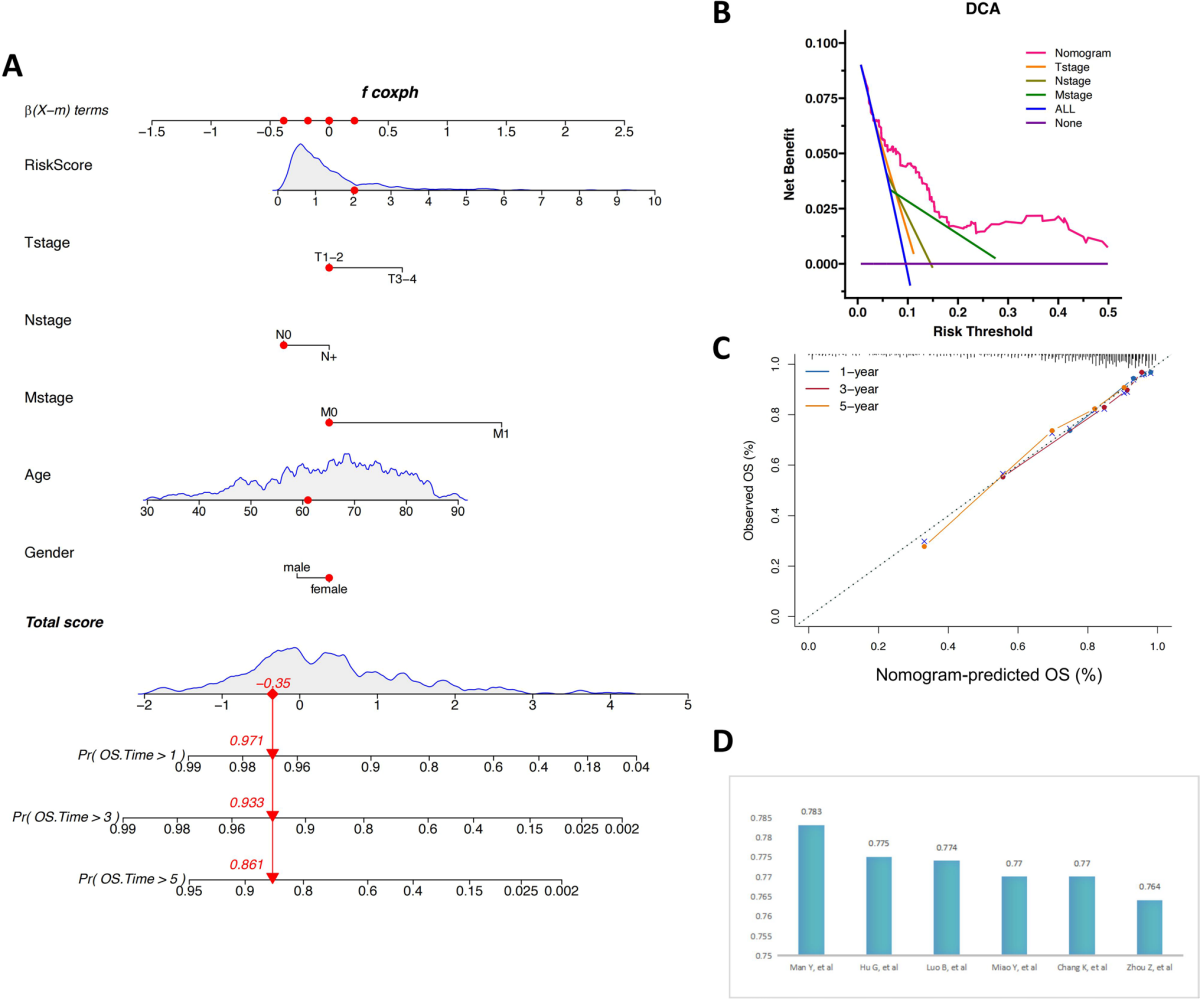
Construction and validation of the nomogram. **A** OS rates of patients for one, three, and five years were predicted by the nomogram constructed with important clinical parameters. **B** DCA evaluated the nomogram compared with the T/N/M stage (one-year DCA). **C** The calibration curves. **D** C-index histogram

A nomogram was constructed based on the prognostic signature related to colorectal MAC-related molecular subtypes. Multivariate Cox regression analysis on potential variables, which included the T/N/M stage, age, gender, and the RiskScore, was used to predict one-, three-, and five-year OS rates in CRC patients (Fig. 10A). The C-index of the nomogram was 0.783. It was observed that a higher RiskScore led to a worse prognosis. For example, a clinician could calculate a 61-year-old female CRC patient with clinical characteristics of T2N0M0 and the RiskScore for a score of the corresponding variables, resulting in a total score of -0.35. The probabilities of survival time longer than one, three, and five years were found to be 0.971, 0.933, and 0.861, respectively.

Calibration curves were also constructed for evaluating the consistency of the OS rates and OS rates predicted by the nomogram. The results showed that this method fit well for one-, three-, and five-year OS prediction (Fig. 10C). In comparison to the T/N/M stage, the results of one-year DCA proved the nomogram had reasonable clinical practicability for prognosis prediction (Fig. 10B). The constructed nomogram was compared with the nomograms of CRC previously published [41–45] and it was found that the nomogram used in this study had a higher C-index (Fig. 10D).

## Discussion

CRC still represents a significant threat to the health of many people. MAC is a unique histopathological subtype of CRC, and its mechanism of occurrence and development, clinical features, and impact on the prognosis of patients still lack understanding. It is urgent to understand the characteristic genes of colorectal MAC in order to guide the clinical precision treatment of CRC.

Previous studies have identified genes with important biological roles for MAC in CRC, such as *RPS18*, *RPL30*, *CXCL9,* and *IDO1* [46, 47]. Machine learning has been applied extensively in biomedicine. Based on the transcriptome data of CRC, this study found significant differences in gene expression between MAC and adenocarcinoma. Functional enrichment analyses showed that MAC-related DEGs were closely related to tumor growth, invasion, or metastasis, which suggested different biological backgrounds between the two histopathological subtypes. Subsequently, three distinct molecular subtypes were distinguished by these MAC-related genes. Further studies were conducted to explore the characteristics of each subtype. There were significant differences in tumor microenvironment, mechanism, and prognosis among different subtypes. These results demonstrated heterogeneity in MAC-related molecular subtypes, and it also provided a direction for our subsequent construction of the risk signature. Based on the MAC-related molecular subtypes of CRC, the signature was constructed in this study, which could perform precise risk stratification of CRC patients.

The signature genes are the highlights of this study. Previous studies have confirmed the important roles of some genes in cancer. In non-small cell lung cancer, the high expression of *CALB1* has a significant correlation with the lymph node metastasis stage [48]. In CRC, MMP1 derived from tumor-associated macrophages promotes the cell cycle transition from G0/G1 to S and G2/M phases [49], and the overexpression of *HOXC6* induces EMT in colon cancer cells [50]. *ZIC2* and *SFTA2* may be identified as critical genes in CRC carcinogenesis [51, 52]. Furthermore, *HYAL1* inhibits CRC metastasis by modulating MMPs/TIMPs balance and rearranging F-Actin distribution [53]. However, these genes have not been studied in depth in the basic research of colorectal MAC and may be the focus of the basic experimental research of colorectal MAC.

This study also analyzed the predictive power of the signature in CRC patients. The C-index of the signature in the TCGA training set, internal testing set, and entire set were 0.683, 0.709, and 0.688, respectively. Based on the signature, a nomogram was constructed by combining the clinical parameters of CRC. The C-index of the nomogram was 0.783. Previous studies established signatures from different perspectives for the prognosis of CRC patients, such as the anoikis-related predictive model [41], the pyroptosis-related predictive model [42], and the RNA-binding protein-related prognostic model [43]. In comparison to the currently published signatures related to CRC, this study built a RiskScore signature based on the MAC-related molecular subtypes. Multiple validations of external datasets were included to improve the reliability. These results suggest that the prognostic signature based on the MAC-related molecular subtypes may enhance the predictive ability of CRC.

The clinical significance and molecular mechanism of the signature were then comprehensively evaluated. It was found that the signature could predict the prognosis accurately and provide a reference for clinical medication. Furthermore, immunotherapy has been studied in full swing in various solid tumors, which brings new hope for CRC. Currently, only CRC patients with MSI-H can benefit from immunotherapy, so a better understanding of the immune microenvironment of CRC wound help to guide immunotherapy strategies. The extracellular mucus protein of MAC can be used as a physical immune infiltration barrier [12]. The complex tumor microenvironment has become a challenge that hinders the treatment of colorectal MAC and leads to the immune escape of CRC. Various tumor microenvironment components are closely related to the occurrence and development of CRC [54]. We were also surprised to find significant differences in infiltrating cells in the tumor microenvironment between different RiskScore groups. Following the further analysis of the molecular mechanism caused by RiskScores, it can be inferred that RiskScores may be engaged in a variety of biological processes leading to different clinical features.

This research still has some limitations. All our data was obtained from the TCGA and GEO databases, and the sample size was not large enough, which may result in a deviation in the results. The exploration of key genes needs to be more profound. Future research should analyze the molecular biological functions of six key genes.

Overall, we performed molecular typing of CRC by analyzing colorectal MAC-related genes. A prognostic signature and the nomogram were then constructed, which was significant for accurate stratification and prognosis evaluation of CRC.

## Data Availability

The datasets provided in this study can be downloaded from online repositories. If you have further inquiries, please contact the authors.

## Abbreviations

AUC: Area under the curve
CI: Confidence interval
CRC: Colorectal cancer
DCA: Decision curve analysis
DEG: Differentially expressed gene
EMT: Epithelial-mesenchymal transition
GEO: Gene Expression Omnibus
GO: Gene ontology
GSEA: Gene set enrichment analysis
GSVA: Gene set variation analysis
HR: Hazard ratio
IC_50_: Half maximal inhibitory concentration
KEGG: Kyoto encyclopedia of genes and genomes
LASSO: Least absolute shrinkage and selection operator
MAC: Mucinous adenocarcinoma
MSI: Microsatellite instability
NMAC: Non-mucinous adenocarcinoma
NMF: Negative matrix factorization
OS: Overall survival
ROC: Receiver operating characteristic
TCGA: The Cancer Genome Atlas
